# HYDEing the false positives – scoring for lead optimization

**DOI:** 10.1186/1758-2946-3-S1-P29

**Published:** 2011-04-19

**Authors:** N Schneider, G Lange, R Klein, C Lemmen, M Rarey

**Affiliations:** 1 University of Hamburg, Center for Bioinformatics, Bundestr 43, 20146 Hamburg, Germany; 2 Bayer CropScience, Industriepark Hoechst, G836, 65926 Frankfurt, Germany; 3 BioSolveIT GmbH, An der Ziegelei 75, 53757 St. Augustin, Germany

## 

It is highly desirable to have a scoring function that provides guidance for the design of compounds with optimized bioactivity. HYDE [1] is such a scoring function, considering the essential interactions in protein-ligand complexes. HYDE describes consistently hydrogen bonds, the hydrophobic effect and desolvation. Its basic principle is a well balanced assessment of the energetics of desolvation. Compared to most other scoring functions HYDE is not calibrated on affinity data, we use octanol/water partition data of small molecules for calibration. Only three major factors are taken into consideration: (a) local hydrophobicity, (b) solvent accessible surface, and (c) contact surface area. Based on these, energetically favorable and unfavorable contributions to the binding affinity can be assessed on an atomic level.

Atomic contributions can be visualized, which turns out to be particularly helpful in a lead-optimization setup. One may immediately identify energetically unfavorable arrangements, like a polar group without a counter-part in an otherwise hydrophobic pocket or two hydrogen bond acceptors facing each other. Medicinal chemists will immediately have ideas how to alter a given structure in order to gain activity.

It is demonstrated that HYDE is able to distinguish between strong binders, weak binders, and non-binders considering several p38 MAP kinase inhibitors [2]. In a congeneric series of thrombin inhibitors [3] it is shown that HYDE is able to score and rank single atom exchanges correctly.
